# An astrocyte cell line that differentially propagates murine prions

**DOI:** 10.1074/jbc.RA120.012596

**Published:** 2020-06-19

**Authors:** Waqas Tahir, Basant Abdulrahman, Dalia H. Abdelaziz, Simrika Thapa, Rupali Walia, Hermann M. Schätzl

**Affiliations:** 1 Department of Comparative Biology & Experimental Medicine, Faculty of Veterinary Medicine, University of Calgary, Calgary, Alberta, Canada; 2 Calgary Prion Research Unit, University of Calgary, Calgary, Alberta, Canada; 3 Hotchkiss Brain Institute, University of Calgary, Calgary, Alberta, Canada; 4 Department of Biochemistry and Molecular Biology, Faculty of Pharmacy, Helwan University, Cairo, Egypt

**Keywords:** prion, prion infection, astrocytes, Creutzfeldt–Jakob disease, scrapie, bovine spongiform encephalopathy, neurodegeneration, protein misfolding, C8D1A, prion disease, astrocyte, neurodegenerative disease, prion strain

## Abstract

Prion diseases are fatal infectious neurodegenerative disorders in human and animals caused by misfolding of the cellular prion protein (PrP^C^) into the pathological isoform PrP^Sc^. Elucidating the molecular and cellular mechanisms underlying prion propagation may help to develop disease interventions. Cell culture systems for prion propagation have greatly advanced molecular insights into prion biology, but translation of *in vitro* to *in vivo* findings is often disappointing. A wider range of cell culture systems might help overcome these shortcomings. Here, we describe an immortalized mouse neuronal astrocyte cell line (C8D1A) that can be infected with murine prions. Both PrP^C^ protein and mRNA levels in astrocytes were comparable with those in neuronal and non-neuronal cell lines permitting persistent prion infection. We challenged astrocytes with three mouse-adapted prion strains (22L, RML, and ME7) and cultured them for six passages. Immunoblotting results revealed that the astrocytes propagated 22L prions well over all six passages, whereas ME7 prions did not replicate, and RML prions replicated only very weakly after five passages. Immunofluorescence analysis indicated similar results for PrP^Sc^. Interestingly, when we used prion conversion activity as a readout in real-time quaking-induced conversion assays with RML-infected cell lysates, we observed a strong signal over all six passages, comparable with that for 22L-infected cells. These data indicate that the C8D1A cell line is permissive to prion infection. Moreover, the propagated prions differed in conversion and proteinase K–resistance levels in these astrocytes. We propose that the C8D1A cell line could be used to decipher prion strain biology.

Prion diseases are a group of fatal and infectious neurodegenerative disorders that include Creutzfeldt–Jacob disease in humans, bovine spongiform encephalopathy in cattle, scrapie in sheep and goat, and chronic wasting disease in cervids. Disease manifestation in human prion disorders can be sporadic, familial, or acquired by infection (1–5), whereas in animals acquired prion diseases prevail (6–9). Pathological hallmarks of prion diseases are vacuolation caused by neuronal death, PrP^Sc^ plaque deposition, astrogliosis, and spongiform degeneration (10). Prion diseases are caused by PrP^Sc^, the pathological isoform of the normal cellular prion protein (PrP^C^), which is highly expressed in neurons, astrocytes, and oligodendrocytes (11). Conformational conversion of PrP^C^ into PrP^Sc^ and self-sustained propagation of PrP^Sc^ are the molecular events driving the disease progression (12–15). There is no treatment available for prion disorders. Although neuronal loss predominates during the clinical manifestation of prion disease (16, 17), the role of non-neuronal cell populations in the CNS in prion pathology cannot be ignored. The presence of PrP^Sc^ aggregates in non-neuronal cell populations in prion-infected mice depleted of neuron-specific PrP demonstrates their potential to propagate prions independent of neuronal PrP (18). Similarly, the susceptibility of astrocyte-specific hamster PrP expressed in PrP knockout mice to hamster scrapie propagation implies a role for astrocytes in prion propagation and infection (19). Differential processing of PrP^Sc^ aggregates by different sets of cells in the CNS could be one of the reasons for the heterogeneity observed in prion pathology. Being a different cell lineage from neurons, astrocytes might process PrP^Sc^ aggregates differently as compared with neurons. However, the exact role of astrocytes in prion propagation and pathogenesis remains elusive. The establishment of various neuronal cell culture models that allowed stable prion infection strongly advanced prion research, provided an experimental platform for studying the cell biology of prion infection, and was used for devising potential anti-prion strategies (20–23). Interestingly, these were mostly established cell lines that allowed persistent propagation of mouse-adapted scrapie prion strains like 22L, RML, or ME7 (24, 25). By using genetically engineered cells, it seems possible to expand the range of prions which can be propagated in such cell lines (26–28). There still is no cell line that can persistently propagate human or bovine prions (25). Non-neuronal cell lines were also very instrumental (29, 30). Of note, with a few exceptions, such cell line models do not show side effects as would be expected from prion pathogenesis *in vivo* (31, 32), an issue that can be better addressed in primary cells (33), cerebral organoids (34), and slice culture models (35). There was a shortage of glia cell models, with ovine microglia cells for propagation of sheep prions (36) and human stem cell–derived astrocytes replicating even human prions (37, 38).

Given the absence of a murine glia-based cell culture model for propagating mouse-adapted prions, we wanted here to characterize an immortalized mouse astrocyte cell line (C8D1A) for its ability to propagate various murine prions (uninfected cells termed C8D and infected cells ScC8D). One of the determining factors for cellular prion propagation is appropriate cell surface expression of PrP^C^ (39). We therefore compared PrP mRNA and surface PrP^C^ expression with that of other cell lines known to stably propagate mouse-adapted prions (N2a cells, CAD5 cells, and mouse embryonic fibroblasts). We then inoculated C8D astrocytes with the prion strains 22L, RML, and ME7 and monitored them for markers of prion infection (immunoblot for PrP^Sc^, PrP^Sc^ immunofluorescence, and prion conversion activity in RT-QuIC) over six passages. Not unexpectedly, astrocytes were not able to sustain persistent infection with all three prion strains but clearly propagated persistent infection with 22L prions as confirmed in all three assays here. Strikingly, astrocytes very differently handled RML prions, with almost no detectable signal in immunoblot and confocal microscopy for PrP^Sc^ but pronounced prion conversion activity over all passages as tested here. This demonstrates that this astrocyte cell line is permissive to prion infection (ScC8D cells) but processes prions very differently regarding prion conversion activity and proteinase K (PK) resistance of PrP. To our knowledge, this is the first report for stable prion infection in immortalized mouse astrocytes. This new cell model could help to advance our understanding of prion strain biology in a non-neuronal context.

## Results

### Comparison of transcriptional and post-translational expression of PrP^C^ in C8D5 astrocytes with other cell lines

**Figure 1.**
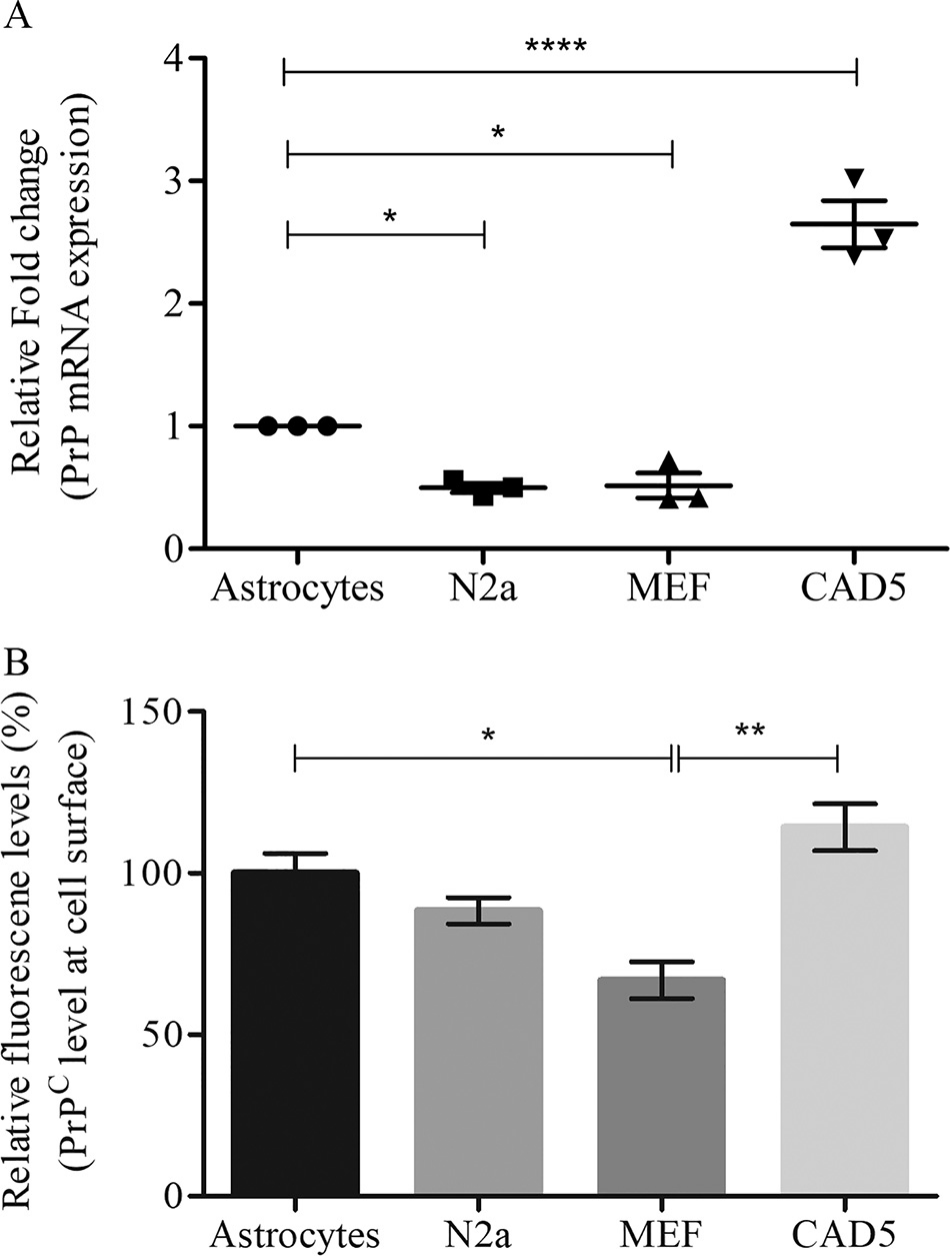
**Expression of PrP^C^ in C8D astrocytes as compared with other neuronal and non-neuronal cell lines.** *A*, mRNA expression of *Prnp* gene in four different cell lines. Relative fold change for PrP mRNA expression was measured by RT-PCR. 1 × 10^6^ cells were plated in triplicate, and after 4 days of culture, total RNA was extracted and reverse-transcribed. cDNA was used for quantitative real-time PCR using SYBR. Relative mRNA expression was normalized with actin and calculated using the ΔΔ*Ct* method. *, *p* value ≤ 0.05 and ***, *p* value ≤ 0.0001 when compared with astrocytes using ANOVA (Dunnett's multiple comparisons) in GraphPad Prism. *Error bars* represent averages ± S.E. *B*, PrP^C^ surface levels in four different cell lines. Surface PrP^C^ was analyzed by FACS after staining with mAb 4H11 and Alexa 405–conjugated secondary antibody. Relative mean fluorescence values of N2a, MEF, and CAD5 were compared with the average fluorescence value of astrocytes, which was set to 100%. Statistical analysis was performed by one-way ANOVA and Dunnett's multiple comparison tests using GraphPad Prism. *, *p* value ≤ 0.05; **, *p* value ≤ 0.01. *Error bars* represent averages ± S.E.

Expression of *Prnp* gene mRNA determines the amount of PrP^C^ present in any cell type. Therefore, we examined the transcriptional expression of *Prnp* in an immortalized mouse neuronal astrocyte cell line (C8D1A) and compared it with PrP mRNA expression in neuronal (N2a and CAD5) and non-neuronal (MEF) cell lines commonly used as *in vitro* prion infection models (20, 40, 41). PrP mRNA levels in astrocytes were ∼2-fold higher than that of N2a and MEF cells but lower than in CAD5 cells (Fig. 1*A*). Because cell surface expression of PrP^C^ is required for establishing prion infection in cultured cells, we next determined the expression of cell surface PrP^C^ in C8D astrocytes and compared it with that of N2a, CAD5, and MEF cells. Our flow cytometry data showed that cell surface expression of PrP^C^ in C8D astrocytes was slightly higher than in N2a cells but lower than that of CAD5 cells, although the differences were not statistically significant. Moreover, cell surface expression of non-neuronal MEF cells was significantly lower than that of astrocytes (Fig. 1*B*). Glial fibrillary acidic protein immunostaining was used to demonstrate the astrocytic cell type (Fig. S1).

Taken together, these data demonstrate that the cell surface expression of PrP^C^ aligns well with mRNA expression in all tested cell lines. Cell surface expression of PrP^C^ in C8D astrocytes is comparable with N2a and CAD5 cells, indicating a potential for prion infection.

### Detection of prion infection in inoculated astrocytes by Western blotting for PrP^Sc^

**Figure 2.**
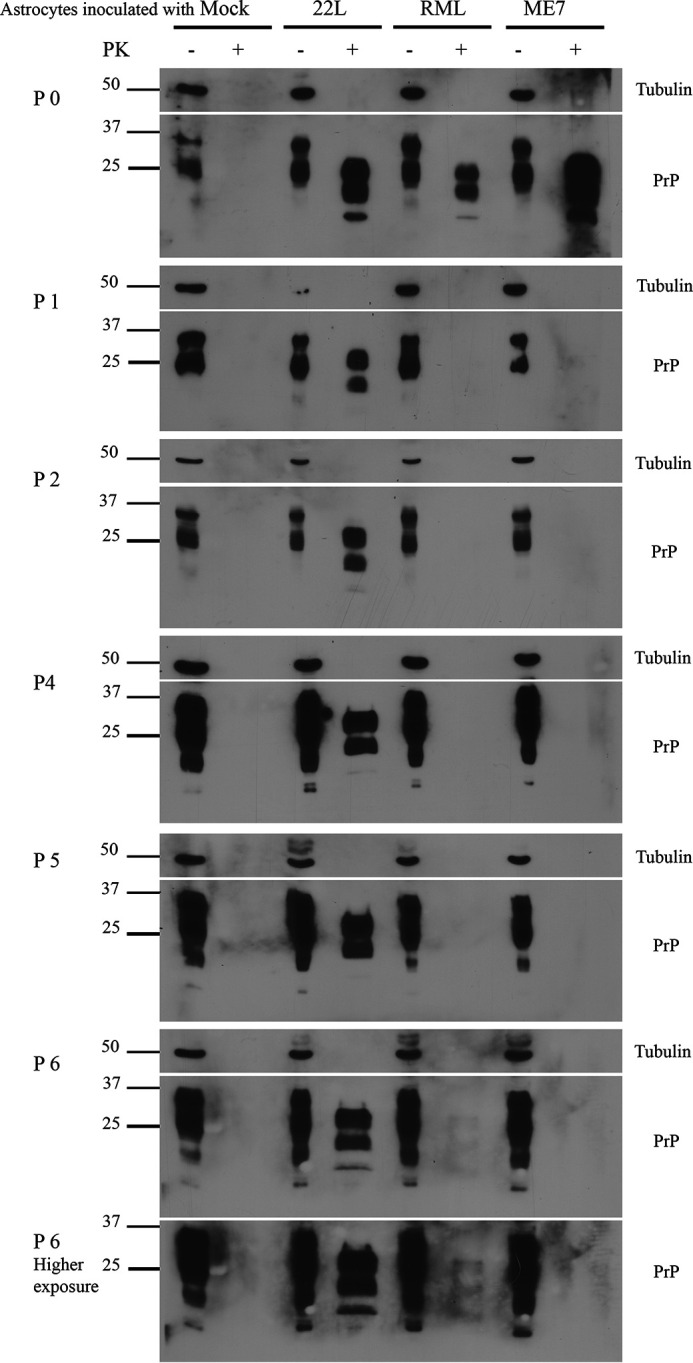
**Levels of PrP^Sc^ in C8D astrocytes infected with 22L, RML, and ME7 prions.** Immunoblots showing PrP^Sc^ levels in astrocytes infected with 22L, RML, and ME7 prions as compared with mock-infected astrocytes (before and after PK digestion). The cells from passage 0–6 were lysed and subjected to PK digestion (+PK) or no PK digestion (−PK). The lysates were immunoblotted and probed with mAb 4H11. α-Tubulin was used as loading control.

Having found that C8D astrocytes express comparable levels of cell surface PrP, we next challenged them with three different murine prion strains (22L, RML, and ME7). Uninfected brain homogenate (mock) was used as a negative control. All brain homogenates were tested by immunoblotting to ensure the presence and comparable levels of PrP^Sc^ (Fig. S2). Astrocytes were passaged six times, and cell lysate aliquots from each passage were analyzed for PK-resistant PrP^Sc^ by immunoblotting. As expected, at passage 0, all astrocyte lysates infected with the three prion strains showed prominent PrP^Sc^ signals, most likely representing residual inoculum (Fig. 2). In passages 1–6, 22L prion-infected astrocytes (ScC8D) maintained PrP^Sc^ in all tested passages, indicative of a persistent prion infection. In contrast, ME7 PrP^Sc^ signal was absent in all subsequent passages (Fig. 2 and Fig. S3). Interestingly, RML prion-infected astrocytes were negative in passages 1–5, and only in passage 6 was a faint PrP^Sc^ signal found (Fig. 2, *high exposure panel*). No PrP^Sc^ was observed in mock-inoculated C8D astrocytes.

**Figure 3.**
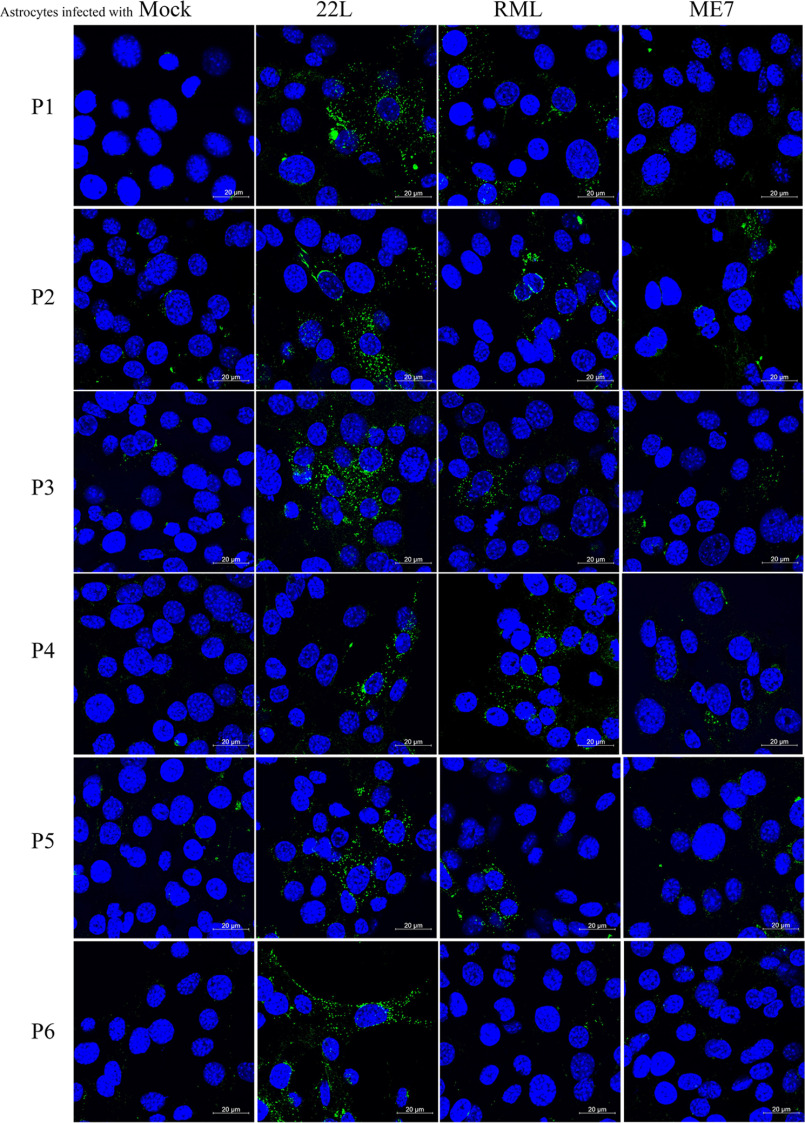
**Immunofluorescence analysis of PrP^Sc^ aggregates in C8D astrocytes infected with 22L, RML, and ME7 prions.** Representative images of PrP^Sc^ immunofluorescence staining in astrocytes infected with 22L, RML, and ME7 prion strains compared with mock-infected astrocytes from passages 1–6. Astrocytes were treated with 6 m guanidine hydrochloride prior to antibody incubation to denature PrP^C^ and retrieve PrP^Sc^ epitopes. PrP^Sc^ was stained with 4H11 as anti PrP antibody (*green*), and the nuclei were counterstained with 4′,6′-diamino-2-phenylindole (*blue*). The cells were visualized by confocal laser scanning microscopy. *Scale bars* represent 20 μm.

These results demonstrate that C8D astrocytes are capable of stably propagating 22L prions. RML prion-infected astrocytes showed PrP^Sc^ at a very late passage only, whereas ME7 prion-infected cells did not show any PrP^Sc^ propagation. These data indicate selective propagation of some murine prion strains over others within the same astrocyte cell line.

### Accumulation of PrP^Sc^ aggregates in prion-infected astrocytes

To further validate our immunoblot results, we used immunofluorescence microscopy for the detection of PrP^Sc^ aggregates in astrocytes. Immunofluorescence analysis involving pretreatment with guanidine salts for epitope retrieval is extensively used for selective detection of PrP^Sc^ (42, 43). Either prion or mock-infected astrocytes were stained with mAb 4H11 after treatment with 6 m guanidine hydrochloride. Confocal laser scanning revealed the presence of typical puncta like granules of PrP^Sc^ aggregates consistently from passage 1–6 in astrocytes infected with the 22L prion strain (Fig. 3). 22L prion-infected astrocytes showed typical perinuclear PrP^Sc^ aggregates as described previously (44, 45), as well as some smaller aggregates distributed all over the cell (Fig. 3 and Fig. S1). RML-infected astrocytes showed weak immunolabeling at passage 3–5 and almost no immunolabeling at passage 6 (Fig. 3). ME7-infected astrocytes provided weak immunolabeling at early passages (P1 and P2), which was fading with passaging.

Taken together, immunolabeling of 22L and ME7 prion-infected C8D astrocytes was in alignment with immunoblotting data. Interestingly, RML-infected astrocytes that started to show PrP^Sc^ in passage 6 in immunoblotting lost their PrP^Sc^ immunolabeling signal over time with almost no signal at passage 6, which is at odds with immunoblotting data.

### Prion conversion activity in C8D astrocytes infected with different prion strains

**Figure 4.**
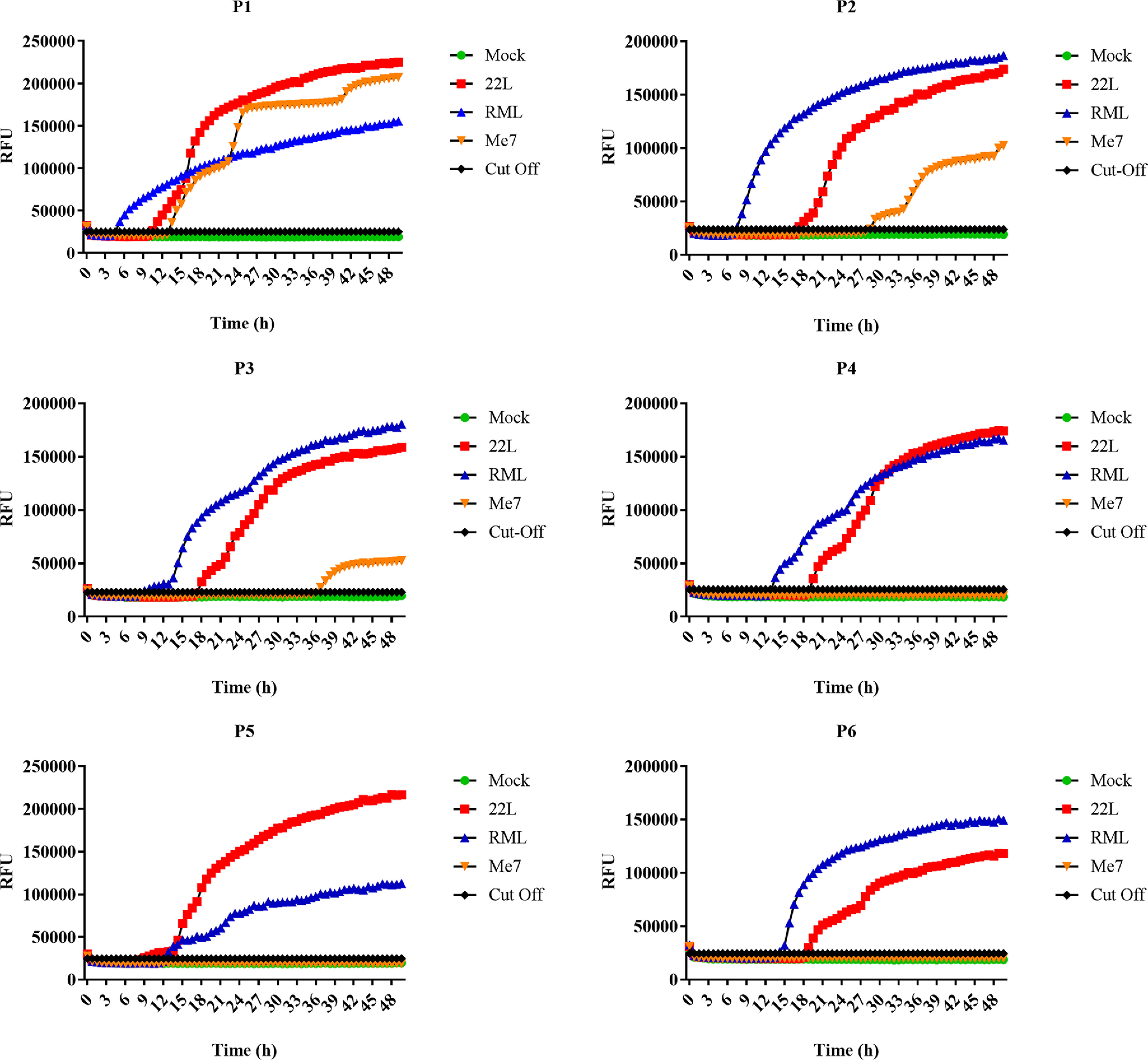
**Prion conversion activity in C8D astrocytes infected with 22L, RML, and ME7 prions.** RT-QuIC assay was performed to determine prion conversion activity in astrocytes infected with 22L, RML, and ME7 prion strains as compared with mock-infected astrocytes. Mouse recombinant PrP was used as substrate. Each reaction was set up in quadruplicate with 2 μl of cell lysate (dilution 10-1 is shown). The average increase of thioflavin-T fluorescence of quadruplicates is plotted as a function of time. The *y* axis represents relative fluorescent units (*RFU*), and the *x* axis shows the time in hours. *A–F*, RT-QuIC analysis of prion-infected astrocytes from passages 1–6 is shown.

Finally, we tested C8D astrocytes for prion conversion activity using real-time quaking-induced conversion (RT-QuIC) assay. This technique assesses prion amplification by monitoring amyloid formation as measured by increase of thioflavin-T fluorescence activity in real time (46). We tested conversion activity of postnuclear lysates of infected astrocytes (22L, RML, or ME7) from P1 to P6. Mock-infected astrocytes from every passage were used as negative control and for calculating the threshold cutoff values. We found that 22L-infected ScC8D astrocytes showed a strong RT-QuIC signature with high RFUs consistently all over the six passages (Fig. 4 and Fig. S4). These results were in accordance with immunoblotting and immunofluorescence data (Figs. 2 and 3, respectively). Surprisingly, RML prion-infected astrocytes that had shown weak and late PrP^Sc^ signals in immunoblotting as well as less PrP^Sc^ aggregates in immunofluorescence experiments revealed very high prion conversion activity in RT-QuIC in all six passages, with five of them becoming positive earlier than 22L prion-infected cells (Fig. 4 and Fig. S4). Of note, the data shown in Fig. S4 were obtained in an independent repeat experiment. As expected, ME7-infected astrocytes showed prion conversion in RT-QuIC assay at the early passages that completely disappeared at passage 4 (Fig. 4), in accordance with immunoblotting and immunofluorescence results (Figs. 2 and 3, respectively).

Taken together, these data indicate that C8D astrocytes propagate the three prion strains very differentially, regarding prion conversion activity and PK resistance and aggregation behavior of PrP^Sc^. Although RML prion-infected cells provided only weak and very late PrP^Sc^ signals in immunoblot, which would grade them almost as noninfected, they showed the highest prion conversion activity over the passages tested here.

**Figure 5.**
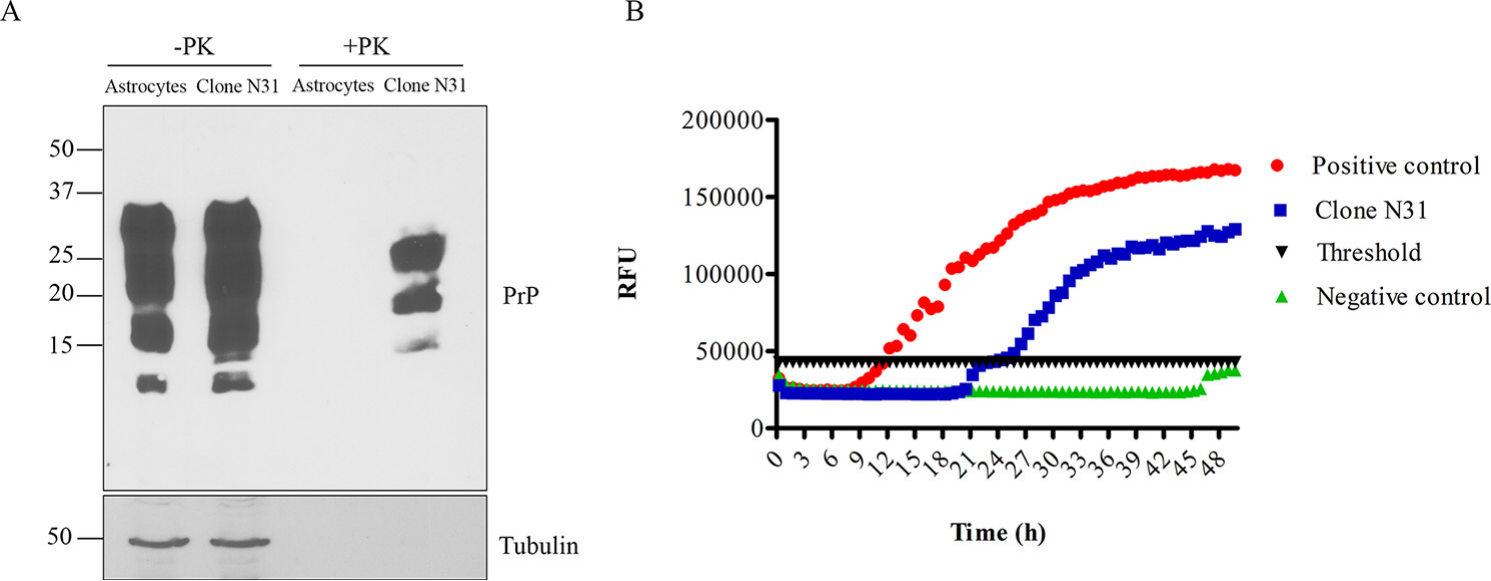
**Stable single-cell clones infected with 22L prions.** *A*, immunoblot analysis showing PrP before (*lanes 1* and *2*) and after PK digestion (*lanes 3* and *4*) in noninfected astrocytes and astrocyte clone N31 persistently infected with 22L prions. Tubulin was used as loading control. *B*, RT-QuIC analysis of the persistently infected astrocyte clone N31. The *y* axis represents relative fluorescent units (*RFU*), and the *x* axis shows the time in hours. Noninfected astrocyte cell lysate was used as negative control, and ScN2a cell lysate was used as positive control. The cells were frozen after passaging for over 6 months for 20 passages after 22L infection. The cells were thawed and cultured again. Immunoblotting and RT-QuIC data represents passage 2 after thawing.

Having obtained a stable PrP^Sc^ signal in 22L-infected astrocytes throughout the six passages, we next cloned them to obtain a persistently infected subclone. The cells were infected newly for this purpose, and single-cell clones were produced by limiting dilution at passage 3. We used RT-QuIC assay for initial screening of persistently infected subclones. Clones that exhibited positive prion conversion were further tested using immunoblotting. Clone N31 demonstrated strong PrP^Sc^ levels in Western blotting and prion conversion activity in RT-QuIC. We continued passaging and testing clone N31 for more than 6 months to verify the persistence of the prion infection (Fig. 5 and Fig. S5). In summary, this new astrocyte cell culture model for prion infection could help to unravel the molecular mechanisms of prion propagation and prion strain biology in glia cells.

## Discussion

Prions are infectious particles composed of misfolded prion protein that can be propagated experimentally both *in vivo* and *ex vivo* with exceptions. The establishment of *in vitro* models of persistent prion infection have greatly enhanced our understanding of the molecular mechanisms of prion formation and propagation, yet many aspects of prion replication remain elusive, including the factors controlling the susceptibility of a given cell line. The permissiveness of a cell line to prion infection is determined by many factors. The endogenous level of expression of PrP^C^ of the same species is important (47), although it is not a requirement for uptake of PrP^Sc^ (48, 49). Differential gene regulatory networks among subclones of a cell line may also facilitate or inhibit permissibility to persistent prion infection (50). On the other hand, prion strains can also exhibit host cell tropism *in vivo* (51, 52) and *ex vivo* (25, 53–57). Furthermore, during the propagation of prions *in vitro*, cell division may reduce the level of PrP^Sc^ by diluting the number of infectious particles over various passages (29). The presence of multiple cell types in the CNS and unique response to prion infection by each of those cell types can further alter the fate of productive prion infection. In this regard, the role of astrocytes and their processing of prions remain elusive.

Astrocytes are a fundamental component of the CNS and represent the most abundant glial cell type in the brain. They are closely associated with neuronal synapses. Astrocytes regulate the transmission of electrical impulses within the brain, provide neurons with nutrients, maintain extracellular ion balance, and play a role in the repair process of brain and spinal cord following traumatic injuries (58–60). It has been reported that chronic neuroinflammation is generally associated with reactive microgliosis and astrogliosis, considered pathological hallmarks of neurodegenerative diseases including Alzheimer's, Parkinson's, amyotrophic lateral sclerosis, and prion diseases (61). How the role and function of astrocytes are altered during prion infection is still controversial, and astrogliosis is mainly considered a secondary event and compensation mechanism to neuronal cell death. On the other hand, several studies showed that prions can propagate in human astrocytes (62), astrocytes of animal origin (47, 63, 64), stem cell–derived astrocytes (38), and astrocytes in mixed primary cultures (65, 66). A growing body of evidence supports that astrocytes impact prion pathogenesis. It has been shown that astrocytes can replicate prions independent of neuronal PrP (67). However, the molecular understanding of the specific role of astrocytes in prion propagation and pathogenesis is still a matter of debate.

Cell culture models are of central importance for studying different aspects of prion propagation and gaining molecular insights into prion biology. Consequently, establishing a cell culture model consisting of stably infected astrocytes would help to understand the exact role of astrocytes in prion propagation better. Currently, there is only one ovine microglia cell line available for propagation of sheep prions (36) and human stem cell–derived astrocytes that can propagate human prions (37, 38). Although most work in prion cell culture was done with mouse-adapted prion strains like 22L, RML, and ME7, there is currently no murine astrocyte cell culture model available that can be persistently infected with mouse prions.

In this study, we describe an immortalized mouse astrocyte cell line that can be infected with 22L and RML prions. Correct expression of PrP^C^ is essential for establishing stable prion propagation in cells, because it is needed as substrate for conversion into PrP^Sc^ (47, 64). It is abundantly expressed in neurons (14, 68). Astrocytes also express PrP^C^ (11), and expression of PrP^C^ in astrocytes was sufficient for inducing prion neurodegeneration (69). However, how PrP^C^ levels compare between astrocytes and other cell lines permissive to prion infection is not known. In this study, we examined PrP^C^ levels at the transcriptional and post-translational level. We compared PrP^C^ levels in C8D astrocytes with levels in a neuronal cell line derived from the peripheral nervous system (N2a), CNS-derived CAD5 cells, and immortalized MEFs. These cells were chosen because of their excellent susceptibility to prion infection (40, 70). Our results revealed that PrP^C^ mRNA levels in C8D astrocytes were comparable with that of N2a and MEF cells but lower than CAD5 cells. Furthermore, the cell surface expression of PrP^C^ in C8D astrocytes was comparable with N2a cells, higher than that of MEFs, and lower than that of CAD5 cells. The rather low expression of cell surface PrP^C^ in MEF cells compared with astrocytes and neuronal cell lines could be explained by the non-neural origin of these cells. From these studies it seemed that the mouse astrocyte line should be a good candidate for prion infection.

Next, we performed infection experiments in C8D astrocytes to establish a mouse glial cell model for stable prion propagation. Of note, we used the uncloned parental astrocyte population for infection experiments with three different prion strains (22L, RML, and ME7). We found that the establishment of a persistent prion infection in C8D astrocytes was prion strain–dependent, similar to findings in other cell lines (21, 41, 54, 56, 70, 71). Immunoblot and immunofluorescence data showed that C8D astrocytes propagated 22L prions very well and stably over all six passages tested. As was expected, this was accompanied by high prion conversion activity as revealed by RT-QuIC analysis. Consequently, single-cell subcloning of 22L-infected astrocytes resulted in a persistently infected clone that showed typical PrP^Sc^ signal in Western blotting and positive conversion activity in RT-QuIC assay, over a culturing period of 6 months. On the other hand, astrocytes inoculated with ME7 prions showed no evidence of persistent PrP^Sc^ propagation. There was a weak prion seeding activity in RT-QuIC analysis in the first passages, which gradually disappeared. We assume this might be due to residual incoming prion inoculum. Alternatively, initial PrP^Sc^ formation might have occurred in the beginning and was not followed by sustained and persistent infection (72). Most surprising were results obtained with RML-infected astrocytes. These cells showed a very late and weak PrP^Sc^ signal in immunoblot, measurable only in passage 6. Similarly, the immunofluorescence pattern for PrP^Sc^ was not very pronounced and much weaker than that of 22L-infected astrocytes, being almost undetectable in passage 6. From these results, the infection in RML-inoculated C8D astrocytes would have been classified as a weak or even borderline infection. Importantly, RML-inoculated astrocytes showed prion seeding activity in RT-QuIC assay comparable with that of 22L-infected astrocytes throughout all six passages. Although RT-QuIC is the most sensitive of the three readouts, these findings were very unexpected. One possibility is that RML-infected astrocytes harbor low amounts of PK-resistant PrP^Sc^ and relatively higher levels of PK-sensitive but infectious PrP. The existence of PrP^Sc^ molecules that are completely degraded by PK and their existence in prion-infected tissues as low-molecular-weight aggregates were described before (73, 74). More recently, Requena and co-workers (75) demonstrated that infectious PK-sensitive PrP shares basic structural features with PK-resistant PrP. In our immunoblot and immunofluorescence analyses, we used harsh PK and guanidine HCl conditions, so PK-sensitive PrP^Sc^ might have been missed. Another possibility is that RML prions are processed very differently from 22L prions in the cultured astrocytes, with higher degradation rates that do not allow accumulation of higher molecular size and more PK-resistant PrP oligomers. How these findings relate to specific prion infectivity in mouse incubation time assays has yet to be seen. Of note, we did not observe major differences between the banding patterns and glycoform ratios (un-, mono-, and diglycosylated PrP^Sc^) between the prion-infected C8D astrocytes and other cell lines tested here for 22L, RML, and ME7 prions.

In summary, our findings suggest that this astrocyte cell line is permissive to prion infection and propagates prions in a strain-specific manner. Whereas strain-specific propagation was previously considered an either/or event, these astrocytes propagate both 22L and RML prions but handle them very differently regarding resistance to proteinase K. This new cell culture system might therefore help to address the biochemical and cell biological determinants of prion strains within a defined cellular background. Unraveling prion strain biology will have implications beyond mammalian prions, because prion-like strain properties are found in many neurodegenerative disorders.

## Experimental procedures

### Reagents and antibodies

Reagents and chemicals were purchased from Sigma–Aldrich, if not otherwise indicated. PK (03115879001) and Pefabloc inhibitor (11286700) were from Roche. The anti-PrP mAb (mAb) 4H11 has been previously described (76). Peroxidase-conjugated secondary antibodies were from Jackson ImmunoResearch (goat anti-mouse HRP and goat anti-rabbit HRP).

### Culture and maintenance of cell lines

The immortalized mouse neuronal astrocyte cell line C8D1A [Astrocyte type I clone] (ATCC^®^ CRL2541^TM^) was purchased from ATCC and was cultured in Dulbecco's modified Eagle's medium GlutaMAX medium (Gibco, 10569-010) containing 10% fetal bovine serum (Sigma, F1051) and 1% penicillin/streptomycin in a 5% CO_2_ atmosphere at 37 °C. The cells were infected overnight with a 1% brain homogenate from terminally sick mice infected with mouse-adapted scrapie prion strains (22L, RML, and ME7). The mouse neuroblastoma cell line N2a was cultured in Opti-MEM GlutaMAX medium (Gibco, 51985-034) containing 10% fetal bovine serum (Sigma, F1051) and 1% penicillin/streptomycin in a 5% CO_2_ atmosphere at 37 °C. CAD5 cells are a central nervous system catecholaminergic cell line (77) and were cultured in Opti-MEM GlutaMAX medium containing 10% bovine growth serum (Hyclone, SH30541.03), and 1% penicillin/streptomycin in a 5% CO_2_ atmosphere at 37 °C. Immortalized MEFs were cultured in Dulbecco's modified Eagle's medium GlutaMAX medium (Gibco, 10569-010) containing 10% fetal bovine serum (Sigma, F1051) and 1% penicillin/streptomycin in a 5% CO_2_ atmosphere at 37 °C.

### RNA extraction, cDNA synthesis, and quantitative real-time PCR

mRNA transcripts of the *PRNP* gene in astrocytes compared with other neuronal as well as non-neuronal cell lines was studied by quantitative real-time PCR. Briefly, equal numbers of cells (1 × 10^6^) for astrocytes, N2a, MEF, and CAD5 cells were cultured in triplicate in 10-cm dishes for 4 days. Total RNA was extracted from cells using an RNeasy mini kit (Qiagen, 74104) according to the instructions of the manufacturer, followed by DNase digestion using RNase-Free DNase set (Qiagen, 79254). The concentration of extracted RNA was measured at 260/280 nm by using a Nanodrop 20000 spectrophotometer (Thermo Fisher Scientific). Extracted RNA was reverse-transcribed into complementary DNA (cDNA) by using a high-capacity cDNA reverse transcription kit (Thermo Fisher Scientific, 4368814) according to the instructions of the manufacturer. Duplicate reactions of each cDNA sample (100 ng) were used in real-time PCR to quantify the relative *PNRP* gene expression using Fast SYBR™ Green Master Mix (Thermo Fisher Scientific, 4385612). The primers used for the *PRNP* gene are: Prnp_F, GTCGCATCGGTGGCAGGACT; and Prnp_R, CAGCCAGTAGCCAAGGTTCGCC (78). Actin was used as a housekeeping gene to normalize the data. The primers used for actin are actin-F (5′-CTCAGGAGGAGCAATGATCTTGAT-3′) and actin-R (5′-TACCACCATGTACCCAGGCA-3′) (79). Real-time PCRs were performed in 96-well PCR plates (VWR, Edmonton, Canada) with a total reaction volume of 20 μl/well using SYBR green dye. The amplification was detected in a CFX96 real-time system C1000 thermal cycler (Bio-Rad). Subsequently, cycle threshold (*Ct*) values were obtained for each sample, and the data were analyzed using the ΔΔ*Ct* method.

### FACS analysis

FACS analysis was used to compare cell surface PrP^C^ expression in astrocytes with that of N2a, MEF, and CAD5 cells. The cells were grown in 6-cm plates until they were confluent, detached from plates using 1 mm EDTA (Sigma-Aldrich), centrifuged, and resuspended in fresh FACS buffer (2.5% FBS in PBS) for 10 min for blocking. 10^5^ cells/staining reaction were incubated with mAb 4H11 primary antibody (1:100) for 45 min, washed three times with FACS buffer, and incubated with goat anti-mouse Alexa Fluor 405 (1:500; Invitrogen, A-31553) secondary antibody for another 30 min. The cells were then washed three times and resuspended in 300 µl of FACS buffer for analysis. All steps were performed on ice and with cold solutions. Flow analysis was performed at the University of Calgary Flow Cytometry Core Facility. All staining reactions were set up in triplicate. Unstained cells and cells stained with secondary antibody alone were used as controls for gating. Monoclonal anti-PrP antibody 4H11 has been described previously (76). Relative mean fluorescence values of N2a, MEF, and CAD5 cells were compared with the average fluorescence value of astrocytes, which was set to 100%. Statistical analysis was performed by one-way ANOVA and Dunnett's multiple comparison tests using GraphPad Prism software. *, *p* values < 0.05; **, *p* values < 0.01. The *bars* represent averages ± S.E.

### Primary infection of astrocytes with prion strains: Preparation of brain homogenates

The mouse-adapted scrapie strains 22L, RML, and ME7 were propagated in C57Bl/6 mice. The mice were euthanized when they were terminally prion sick, and the brains extracted under sterile conditions. The brain homogenates were prepared by homogenizing brains in PBS (10% w/v) using a gentle MACS^TM^ dissociator for 2 min at room temperature, succeeded by centrifugation at 2,000 × *g* for 1 min, then aliquoted, and stored at −80°C until further use. All brain homogenates used for astrocyte infection experiments were also tested by Western blotting for (−PK) and (+PK) (50 µg/ml final concentration for 1 h) PrP to confirm the presence of PrP^Sc^ in brain homogenates.

### Prion infection of astrocytes

Primary prion infection of C8D astrocytes was done by seeding 1 × 10^5^ cells in a 12-well culture plate for 24 h. On the next day, the culture medium was removed, and the cells were overlaid with 50 μl of 10% brain homogenate (22L, RML, ME7, or mock) in 450 μl of serum-free culture medium. After 5 h of incubation, 500 μl of complete culture medium was added. After 24 h, medium was removed, and the cells were washed once with PBS followed by the addition of fresh culture medium to the cells. The cells were passaged several times, and cell lysates were collected from each passage for subsequent analysis for the presence of PrP^Sc^ in immunoblot and analysis of seeding activity in RT-QuIC assay.

### PK digestion and immunoblotting of prion-infected astrocytes

PK digestion and immunoblotting were performed as previously described (80). Briefly, confluent astrocytes were washed with PBS followed by incubation with cold lysis buffer (10 mm Tris-HCl, pH 7.5, 100 mm NaCl, 10 mm EDTA, 0.5% Triton X-100, 0.5% sodium deoxycholate) for 10 min. Lysed astrocytes were centrifuged for 14,000 rpm for 1 min, and supernatant was collected. 500 µl of supernatant was incubated either with PK (final concentration, 20 µg/ml) at 37 °C for 30 min for samples with PK digestion (PK+) or with proteinase inhibitors for samples without PK treatment (PK−). PK digestion was stopped by addition of proteinase inhibitors (0.5 mm Pefabloc) followed by direct methanol precipitation of both PK+ and PK− samples at −20 °C overnight. Precipitated proteins were resuspended in TNE buffer (50 mm Tris-HCl, pH 7.5, 150 mm NaCl, 5 mm EDTA) and 3× sample loading buffer. The samples were run on 12.5% SDS-PAGE, electroblotted on Hybond P 0.45 polyvinylidene difluoride membrane (Amersham Biosciences, 10600023), and immune reactivity was detected by using Luminata Western chemiluminescent HRP substrates (Millipore, WBLUF0100).

### Immunofluorescence and confocal imaging

For immunofluorescence-based detection of PrP^Sc^ aggregates in astrocytes, 1 × 10^5^ cells of prion-infected (22L, RML, and ME7) astrocytes were seeded in 12-well culture plate. Once 60–70% confluent, the cells were fixed with 4% paraformaldehyde for 30 min at room temperature, followed by quenching of autofluorescence (with 50 mm NH_4_Cl, 20 mm glycine) for 10 min at room temperature. The cells were permeablized with PBS containing 5% FBS and 0.3% Triton X-100 for 30 min followed by denaturation of PrP^C^ and epitope retrieval of PrP^Sc^ by incubation with 6 m guanidine HCl for 10 min at room temperature as described previously (44, 45). The cells were incubated with anti-PrP mAb 4H11 (diluted at 1:100 in PBS containing 5% FBS and 0.3% Triton X-100) overnight at 4 °C. Alexa Fluor 488 goat anti-mouse secondary antibody (Jackson Immunoresearch) was used (at 1:200 in PBS containing 5% FBS and 0.3% Triton X-100) for 1 h at room temperature to visualize immunofluorescence. 4′,6′-Diamino-2-phenylindole (1:5000 in PBS) was used as a nuclear stain. Glial fibrillary acidic protein rabbit pAb was purchased from DAKO (Z0334). All images were captured under 63× oil lens at the same acquisition settings from a Zeiss LSM 700 confocal microscope.

### Real-time quaking-induced conversion (RT-QuIC) assay

#### Preparation of recombinant protein

Recombinant prion protein was prepared as described previously (46, 81). Briefly, mouse PrP with amino acid sequence 23–231 was cloned into pET-41 plasmids and transformed into *Escherichia coli* Rosetta, and bacteria was cultured in LB medium in the presence of kanamycin (0.05 mg/ml) and chloramphenicol (0.034 mg/ml). Protein expression was induced using an Overnight Express autoinduction system (Novagen, 71300) followed by isolation of inclusion bodies from pelleted cells using Bug Buster Master Mix (Novagen, 71456) and storage at −20°C. To purify recombinant PrP, inclusion bodies were solubilized in guanidine buffer (8 m guanidine HCl, 100 mm sodium phosphate, 10 mm Tris-HCl, pH 8.0) followed by incubation on a rocker for 1 h at room temperature. Nickel–nitrilotriacetic acid Superflow resin beads (Qiagen, 1018401) were incubated in denaturing buffer (6 m guanidine HCl, 100 mm sodium phosphate, pH 8.0) for 1 h at room temperature. Once solubilized, inclusion bodies were centrifuged at 16,000 × *g* for 5 min, the supernatant was collected, added to the beads, and incubated with gentle rocking. After 1 h, the beads were packed into a XK16 glass column (GE Healthcare Life Sciences; 28988937; length, 200 mm) and attached to an AKTA Explorer chromatography system for protein purification at room temperature. Protein was refolded with a gradient from 100% denaturing buffer to 100% refolding buffer (100 mm sodium phosphate, 10 mm Tris-HCl, pH 8.0) for 4 h by using an Amersham Biosciences ÄKTA Explorer FPLC unit running with Unicorn software (5 version, GE Healthcare Life Sciences). The central portions of the A280 UV peak were collected into dialysis buffer (10 mm sodium phosphate, pH 5.8). Purified protein was filtered with a 0.22-μm filter followed by transfer into a Slide-A-Lyzer dialysis cassette (molecular mass, 10 kDa; Thermo Scientific, 87731) and placed in a beaker with dialysis buffer with continuous stirring for overnight at 4 °C. After dialysis, a prewashed 0.22-μm Argos syringe filter was used to filter the protein solution again followed by measurement of protein concentration with BCA protein assay (Thermo Scientific, 23227). Finally, the protein solution was aliquoted and stored in −80°C until further use.

#### RT-QuIC assay

The RT-QuIC assay was done as described previously (81). The master mix (98 μl/well) containing a final concentration of 10 mm phosphate buffer (pH 7.4), 300 mm NaCl, 0.1 mg/ml rPrP, 10 μm ThT, and 1 mm of EDTA was dispensed in the wells of a 96-well optical bottom plate (Nunc, 165305). Cell lysate dilutions (2 μl) from prion-infected (22L, RML, or ME7) and corresponding mock-inoculated astrocytes was seeded into corresponding wells to a final reaction volume of 100 μl/well. Each reaction was carried in quadruplicate and contained a final concentration of 0.002% SDS. The plates were sealed with Nunc amplification tape (Nalge Nunc International) and incubated in FLUOstar Omega (BMG Labtech, Cary, NC) at 42 °C for 50 h with each cycle comprised of shaking at 700 rpm for 60 s followed by rest for 60 s throughout the incubation. Measurements of ThT fluorescence (450-nm excitation and 480-nm emission; bottom read) were taken every 15 min. RT-QuIC data were analyzed by taking the average of four replicate wells and normalizing to a percentage of the maximum fluorescence response of the instrument. The average values were plotted against the reaction times. Cutoff was calculated as five times the standard deviation of the mean of reaction values of mock samples. The samples were considered positive for prion seeding activity if at least two of the quadruplicates reached the ThT fluorescence cutoff.

### Astrocytes subcloning

Astrocytes were seeded at 1 × 10^5^ cells in a 12-well culture plate for 24 h. The next day, the culture medium was removed, and the cells were overlaid with 50 μl of a 10% 22L brain homogenate in 450 μl of serum-free culture medium. After 5 h of incubation, 500 μl of complete culture medium was added. After 24 h, medium was removed, and the cells were washed once with PBS followed by addition of fresh culture medium to the cells. The cells were passaged two times, then cells were trypsinized, and the cell suspension was diluted to the equivalent of one cell per well and transferred to 96-well plates for limiting dilution single-cell cloning. After 10–15 days, single-cell clones were picked and transferred to larger plates. Subclones were initially tested by RT-QuIC assay for detecting their prion conversion activity. All the subclones that showed positive prion conversion activity were tested by immunoblotting to confirm the presence of *bona fide* PrP^Sc^. We followed up the prion infection in the successful subclone for more than 6 months to ensure the persistence of the prion infection.

### Statistical analysis

Statistical analysis was performed by one-way ANOVA followed by post Dunnett's multiple comparison tests using GraphPad Prism software. *, *p* values < 0.05; **, *p* values < 0.01. *Bars* represent averages ± S.E. The graphs were plotted using GraphPad Prism 7.

## Data availability

All data in this study are contained within the article.

## Supplementary Material

Supporting Information
