# Supplementary material for: An astrocyte cell line that differentially propagates murine prions
Source: J Biol Chem. 2020 Jun 19;295(33):11572–83. doi: 10.1074/jbc.RA120.012596 (PMC7450132; doi:10.1074/jbc.RA120.012596)
Supplement: Supporting Information [file supp_295_33_11572__index.html]

An astrocyte cell line that differentially propagates murine prions — Differential propagation of mouse prions in astrocytes — An astrocyte cell line that differentially propagates murine prions — Differential propagation of mouse prions in astrocytes — Supporting Information 

# An astrocyte cell line that differentially propagates murine prions

## Supporting Information

- Supporting Information (to be published online) - Supporting information
