# Supplementary material for: An astrocyte cell line that differentially propagates murine prions
Source: J Biol Chem. 2020 Jun 19;295(33):11572–83. doi: 10.1074/jbc.RA120.012596 (PMC7450132; doi:10.1074/jbc.RA120.012596)
Supplement: Supporting Information [file supp_RA120.012596_158154_2_supp_550924_qc4yrw.pdf]

An astrocyte cell line that differentially propagates murine prions

Waqas Tahir, Basant Abdulrahman, Dalia H. Abdelaziz, Simrika Thapa, Rupali Walia, Hermann M. Schatzl

S-1

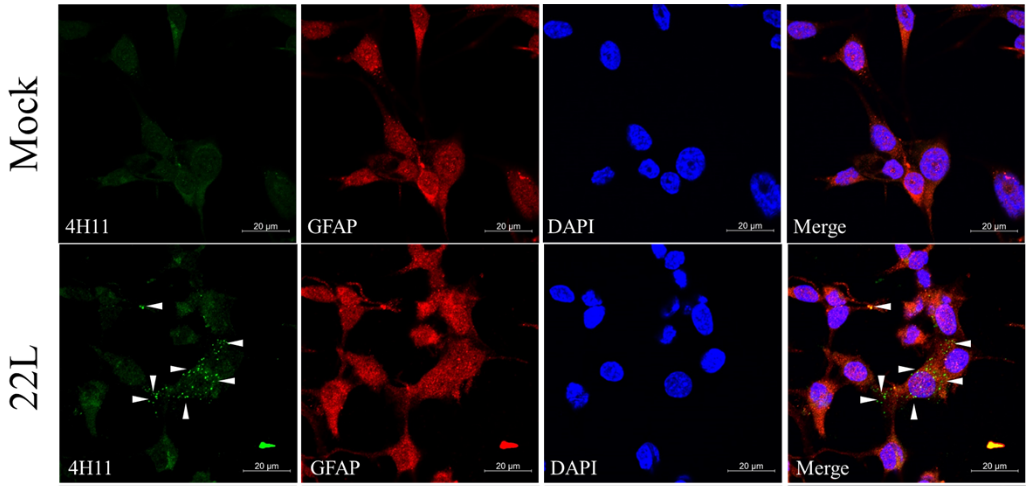

Figure S1: Double immunofluorescence of C8D astrocytes infected with 22L prion strain. Double staining was performed by using GFAP to map astrocytic structures and 4H11 to map PrP^{Sc} aggregates in astrocytes. Astrocytes were treated with 6M guanidine hydrochloride prior to antibody incubation to denature PrP^{C} and retrieve PrP^{Sc} epitopes. PrP^{Sc} was stained with 4H11 as anti PrP antibody (green), astrocytes were stained with GFAP (red), and nuclei were counterstained with DAPI (blue). PrP^{Sc} positive staining is represented by white arrowheads in 22L infected astrocytes. PrP^{Sc} aggregates were found localized in peri-nuclear regions as well as throughout the whole the processes of astrocytes. The cells were visualized by confocal laser scanning microscopy. Scale-bars represent 20 µm.

S-2

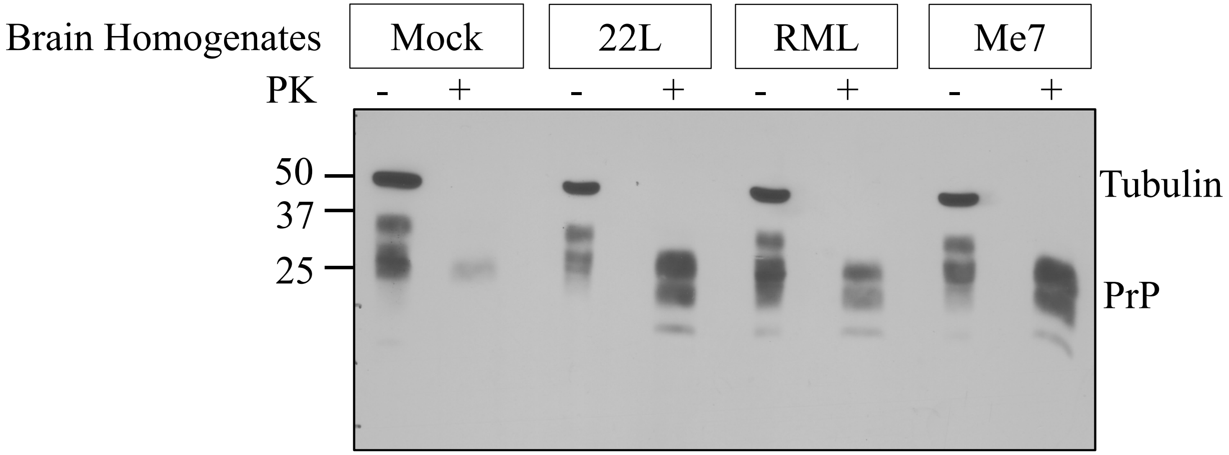

Figure S2: Levels of PrP^{C}/PrP^{Sc} in the brain homogenates (with and without PK digestion) of mock, 22L, RML and ME7 infected terminal sick mice used as inoculum for *in vitro* prion infection of astrocytes. Fifty µg/ml of PK was used for PK digestion. Tubulin was used as a loading control.

S-3

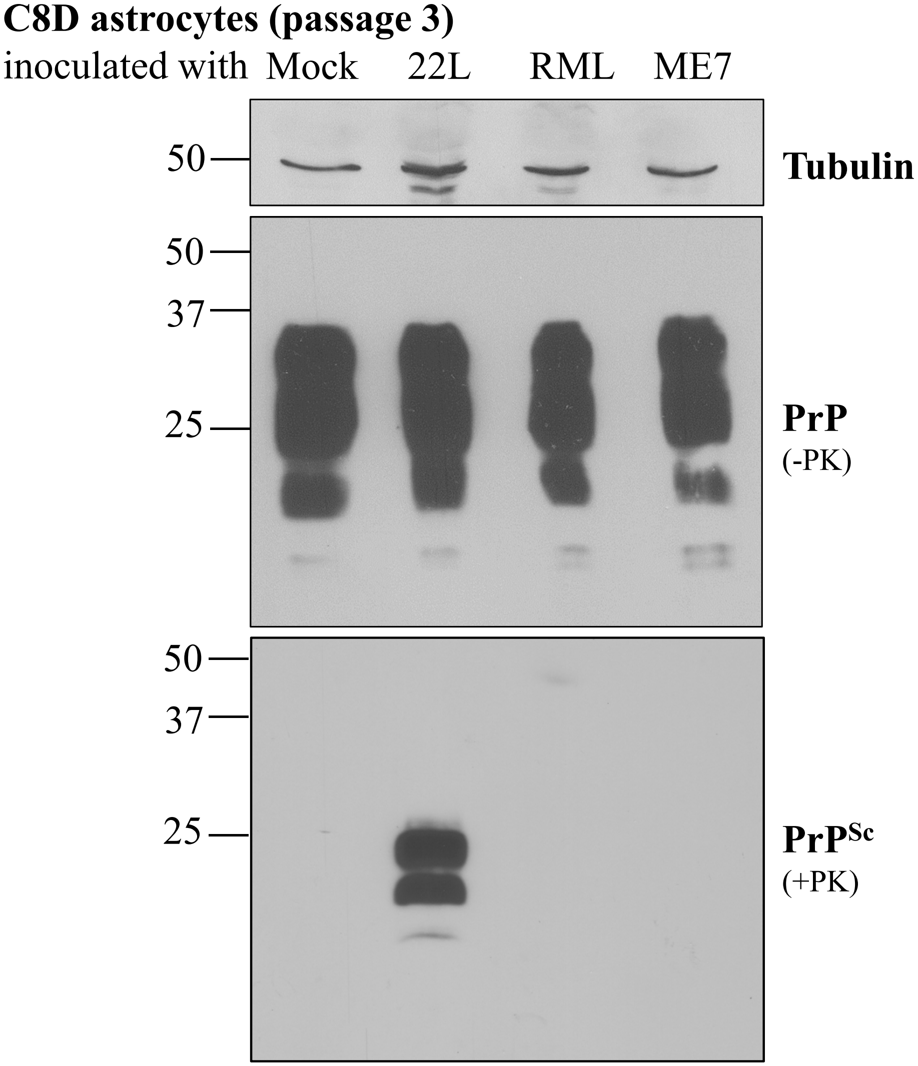

Figure S3: Levels of PrP in C8D astrocytes infected with 22L, RML and ME7 prions in passage 3. Cells were lysed at passage 3 and subjected to PK digestion (+PK) or no PK digestion (-PK). The lysates were separated on SDS-PAGE and immunoblotted with mAb 4H11 (1:10,000) and immuno-reactivity detected with luminata western chemiluminescent HRP substrate. Tubulin was used as loading control. Only 22L-infected astrocytes showed PK resistant PrP.

S-4

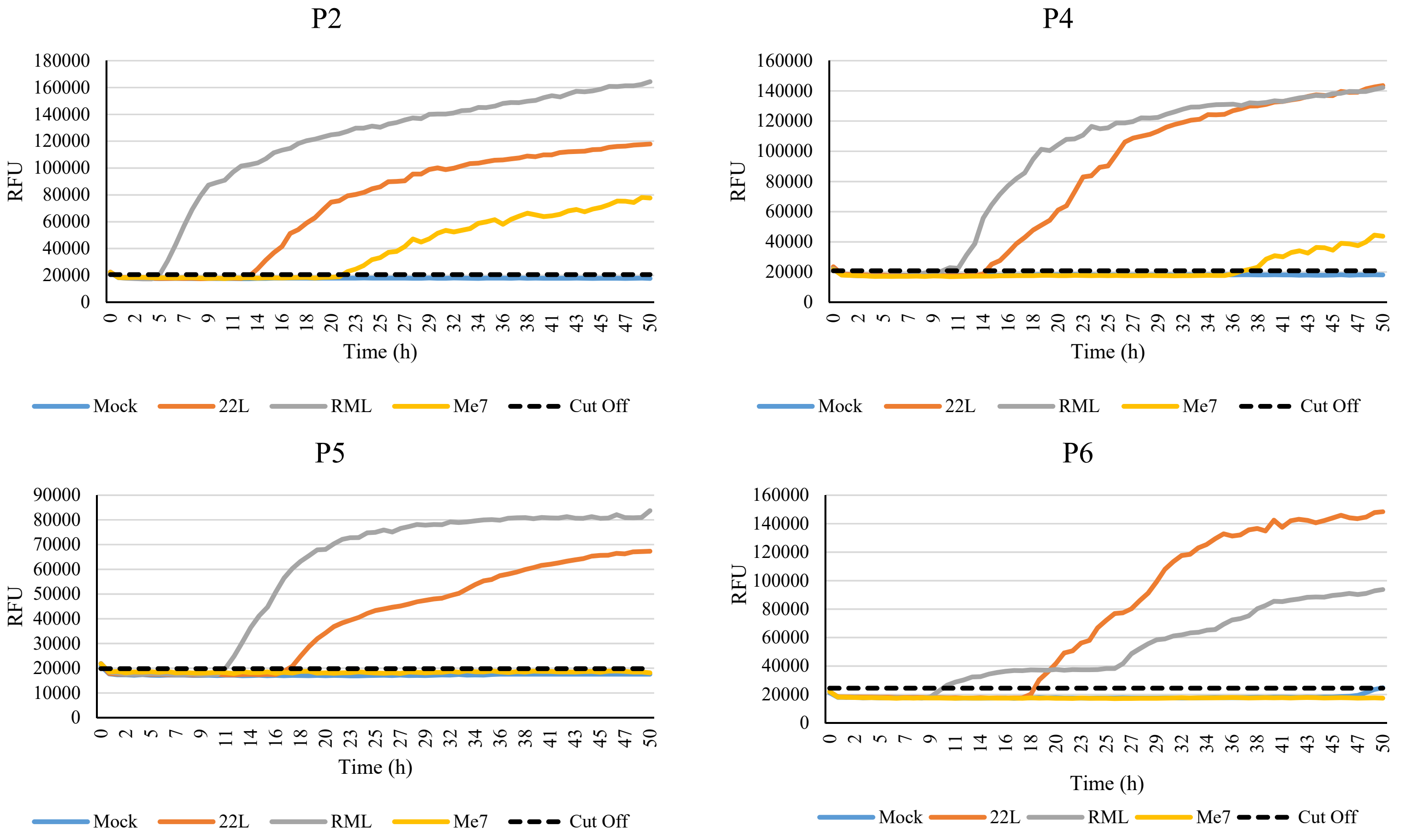

Figure S4: Prion conversion activity in C8D astrocytes infected with 22L, RML and ME7 prions. Infection experiment was repeated and infected astrocytes were again cultured for 6 passages. RT-QuIC assay was performed to asses prion conversion activity, using mouse recombinant PrP as substrate. Each reaction was set up in quadruplicates with 2 µl of cell lysate (dilution: 10^{-1}). The average increase of Thioflavin-T fluorescence of quadruplicates is plotted as a function of time. Y-axis represents relative fluorescent units (RFU) and x-axis time in hours. RT-QuIC analysis from passage 2, 4, 5 and 6 is shown.

S-5

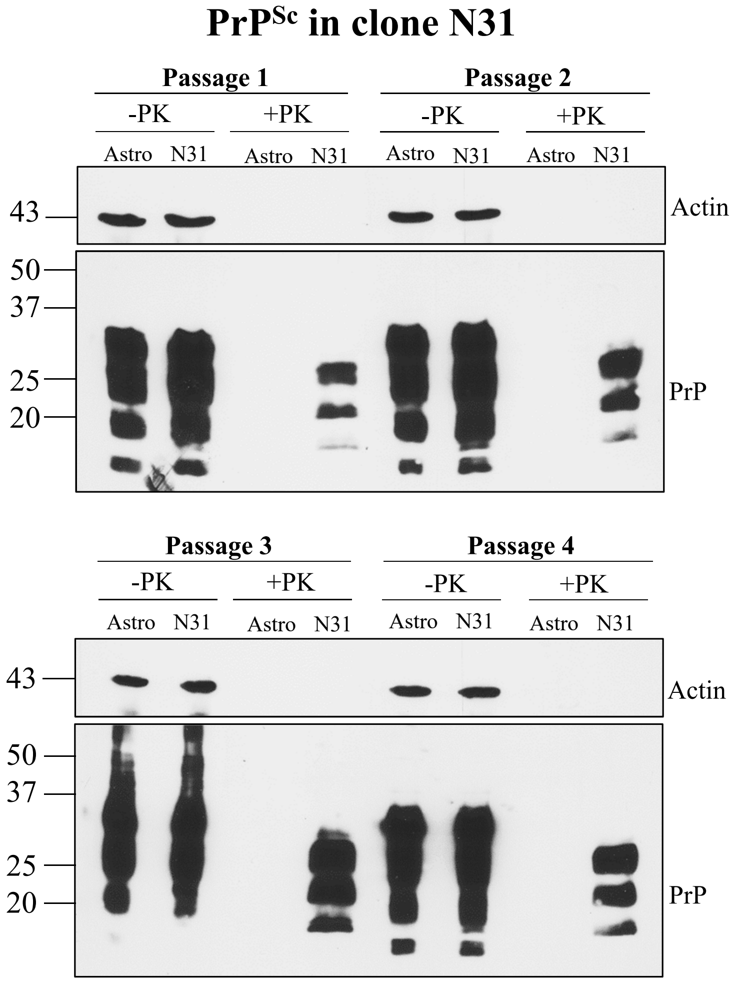

Figure S5:. Effect of freezing and thawing on PrP^{Sc} levels in astrocyte clone N31 persistently infected with 22L prions. Clone N31 was frozen and re-cultured after three months followed by culturing for four passages. Immunoblots show PrP^{C}/PrP^{Sc} levels in non-infected C8D astrocytes (astro) and clone N31 (N31) from passage 1-4 before and after PK digestion (20 µg/mL). The lysates were separated on SDS-PAGE, immunoblotted with mAb 4H11 (1:10,000) and immuno-reactivity was detected with luminata western chemiluminescent HRP substrate. Actin was used as loading control. Astrocyte clone N31 showed PrP^{Sc} persistently from passage 1-4. Please note that the blot for Passage 2 from this experiment was used intentionally for Fig. 5A in the main manuscript. Experiments depicted in Fig. S5 demonstrate presence of PrP^{Sc} in the persistently infected clone N31 at comparable levels over 4 passages after re-culturing cells, Fig. 5 the presence of PrP^{Sc} *per se* in the clone.
